# Poor Functional Outcome Following Total Knee Replacement Due to Underlying Lumbar Canal Stenosis: A Case Report

**DOI:** 10.7759/cureus.105668

**Published:** 2026-03-22

**Authors:** GM Shafeeq, Mohammed Faizan, Paresh Bang

**Affiliations:** 1 Spine Surgery, Max Super Speciality Hospital, Nagpur, IND

**Keywords:** lumbar canal stenosis, lumbar decompression surgery, neurogenic claudication, persistent postoperative pain following tkr, total knee replacement

## Abstract

The current era of orthopedic practice is marked by increasing subspecialization. Many surgeons concentrate primarily on conditions within their specific area of expertise, which can inadvertently narrow their clinical perspective. As a result, patients may not always be evaluated holistically, and this limitation can lead to the misdiagnosis of the underlying primary condition. Total knee replacement (TKR) is an effective treatment for advanced osteoarthritis; however, persistent postoperative lower limb pain may indicate an alternative diagnosis. Pre-existing lumbar canal stenosis (LCS) is one common cause. Overlapping symptomatology between knee pathology and spine problems can lead to misdiagnosis and suboptimal surgical outcomes.

We report a 59-year-old woman with bilateral lower limb pain for two years, worsening over the preceding three months. She underwent bilateral TKR for radiologically confirmed severe osteoarthritis in an arthroplasty center but continued to experience disabling leg pain and poor functional improvement despite structured rehabilitation. Evaluation at our spine clinic revealed neurogenic claudication with limited walking tolerance, preserved prosthetic alignment, and significant stiffness of both knees. Magnetic resonance imaging (MRI) of the lumbar spine demonstrated severe L4-L5 LCS. Following lumbar decompression surgery, the patient experienced substantial relief of leg pain and neurogenic symptoms, enabling improved participation in knee rehabilitation.

This case illustrates how unrecognized LCS can contribute to persistent postoperative symptoms and poor functional recovery following TKR. Lumbar decompression effectively resolved the patient's neurogenic symptoms, confirming spinal pathology as the primary cause of persistent pain rather than prosthetic complications.

Persistent lower limb pain after TKR warrants a thorough evaluation of other causes of pain including lumbar spine pathology. Early recognition and treatment of LCS are essential to optimize postoperative outcomes and prevent prolonged dysfunction. Spine assessment should be integral to the preoperative planning of TKR especially in patients with atypical or disproportionate symptoms.

## Introduction

Modern orthopedic practice has entered a phase of pronounced subspecialization. While focused expertise offers many benefits, it also means that surgeons may concentrate predominantly on their specific domain, sometimes at the expense of a comprehensive patient assessment. Consequently, this fragmented approach can increase the risk of overlooking or misdiagnosing the primary underlying pathology. A similar clinical scenario was encountered a few months earlier in a patient with concurrent knee and spinal pathology. This case report aims to highlight the importance of careful clinical evaluation in order to avoid such diagnostic oversights in future practice.

Total knee replacement (TKR) is widely regarded as an effective surgical intervention for end-stage osteoarthritis, offering substantial pain relief and functional improvement in most patients. However, persistent postoperative lower limb symptoms, particularly when disproportionate to radiological findings, should prompt evaluation for alternative etiologies. Lumbar spine pathology, especially lumbar canal stenosis (LCS), can mimic or exacerbate knee pain and is often overlooked in the preoperative assessment of TKR candidates.

LCS is characterized by narrowing of the spinal canal, resulting in the compression of the nerve roots or cauda equina [1]. Compression of the lumbar nerve roots, particularly L3 and L4, may produce referred pain to the anterior thigh and knee, thereby mimicking primary knee pathology [2]. Patients may present with anterior knee pain in the absence of significant intrinsic knee pathology, pain radiating from the lower back to the knee, neurogenic claudication characterized by leg pain during walking that is relieved by sitting or lumbar flexion, and quadriceps weakness secondary to L3-L4 nerve root involvement [1,3]. Because the L3-L4 dermatomal distribution includes the anterior knee region, patients may predominantly complain of knee pain rather than back pain, which can lead to diagnostic confusion and potential misdiagnosis [2,4].

Studies have shown a high prevalence of coexisting lumbar degenerative spine disease in elderly patients undergoing TKR, with up to 54-68% demonstrating radiographic lumbar spine abnormalities and 30% reporting persistent symptoms post-TKR attributable to spinal pathology [5,6]. Neurogenic claudication due to LCS can cause lower limb pain, fatigue, and functional limitation that may be misattributed to knee arthritis [3]. Failure to identify and address LCS preoperatively may compromise postoperative rehabilitation and overall functional outcome after TKR.

We present a case of a 59-year-old woman with persistent lower limb pain following bilateral TKR, ultimately diagnosed with severe L4-L5 LCS with instability and successfully treated with lumbar decompression and fusion.

## Case presentation

**Figure 1:**
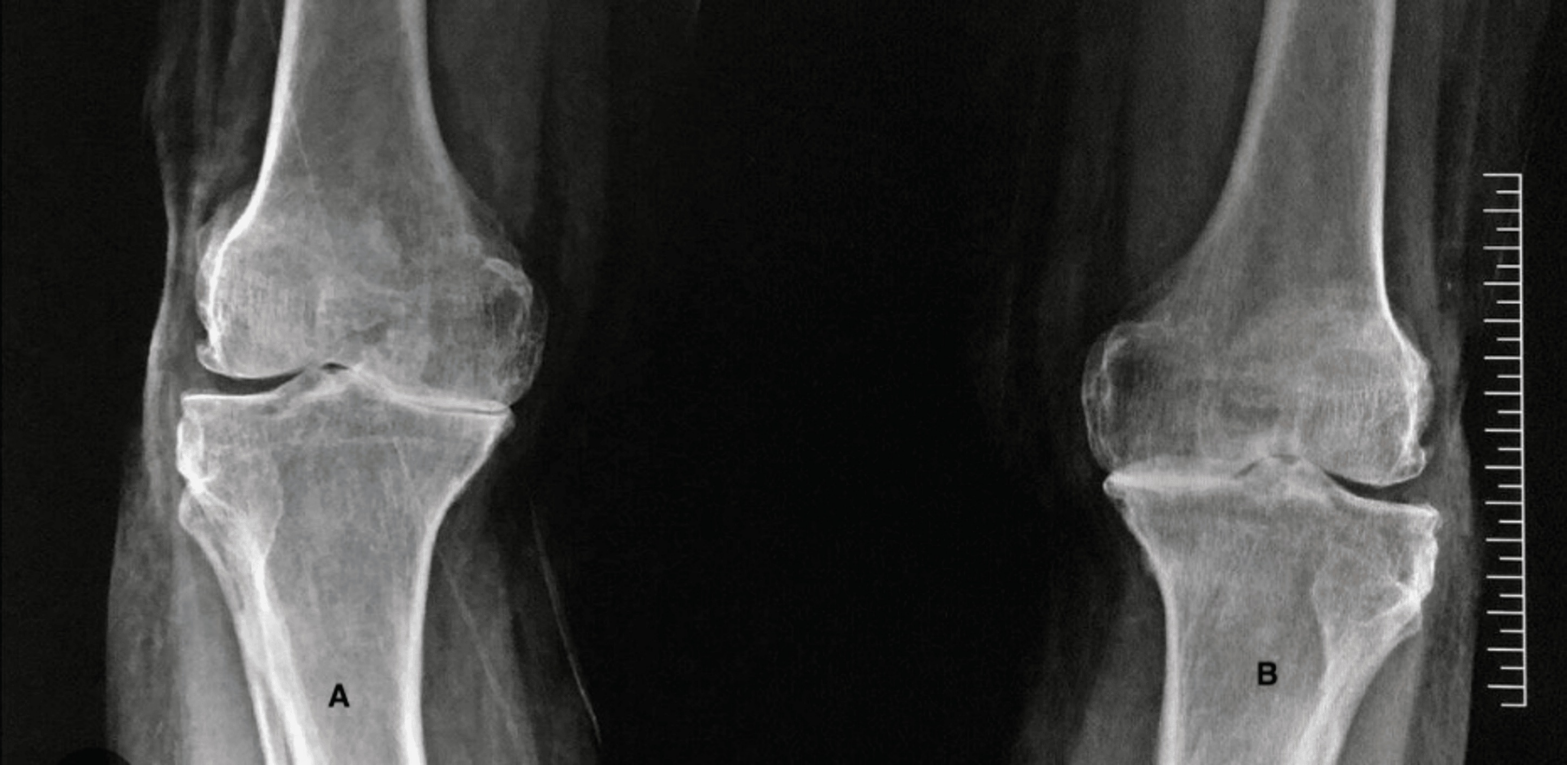
X-ray standing AP view showing severe osteoarthritis in both knee joints (A) X-ray standing AP view of the right knee showing severe osteoarthritis mainly in the medial joint compartment with varus deformity. (B) X-ray standing AP view of the left knee showing severe osteoarthritis mainly in the medial compartment with varus deformity. AP: anteroposterior

A 59-year-old woman presented with bilateral lower limb pain for two years, which had progressively worsened over the preceding three months. The patient reported severe knee pain with significant limitation in ambulation, being unable to walk more than 10 meters. Notably, due to the predominance of knee pain, she did not report significant low back pain or intermittent claudication of the lower limbs. Initial evaluation was performed at an arthroplasty center. According to the previous medical records, clinical examination revealed severe bilateral knee joint line tenderness and restricted range of motion secondary to pain, while motor and sensory examinations were normal. Bilateral knee radiographs demonstrated features consistent with severe osteoarthritis (Figure 1). 

**Figure 2:**
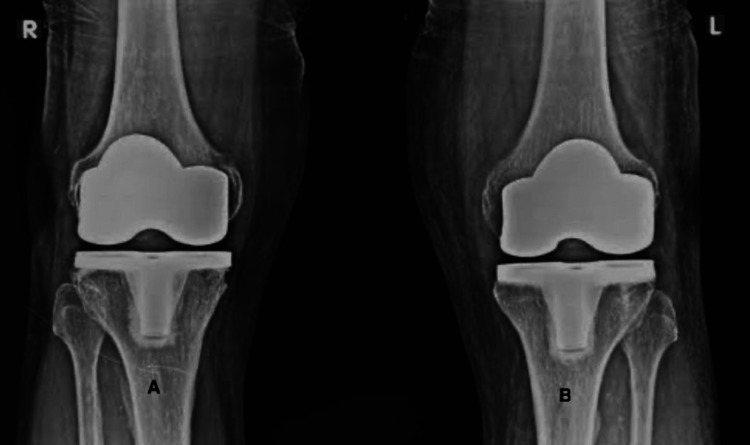
X-ray standing AP view of both knees showing bilateral TKR prostheses in good alignment (A) X-ray standing AP view of the right knee showing TKR prosthesis in good alignment. (B) X-ray standing AP view of the left knee showing TKR prosthesis in good alignment. AP: anteroposterior; TKR: total knee replacement

The patient subsequently underwent bilateral TKR performed by an arthroplasty surgeon (Figure 2).

Despite appropriate postoperative care, including structured physiotherapy and a standard TKR rehabilitation protocol for one month, the patient reported minimal improvement in her leg pain and difficulty ambulating. She was reassured initially but sought further evaluation due to persistent symptoms.

**Figure 3:**
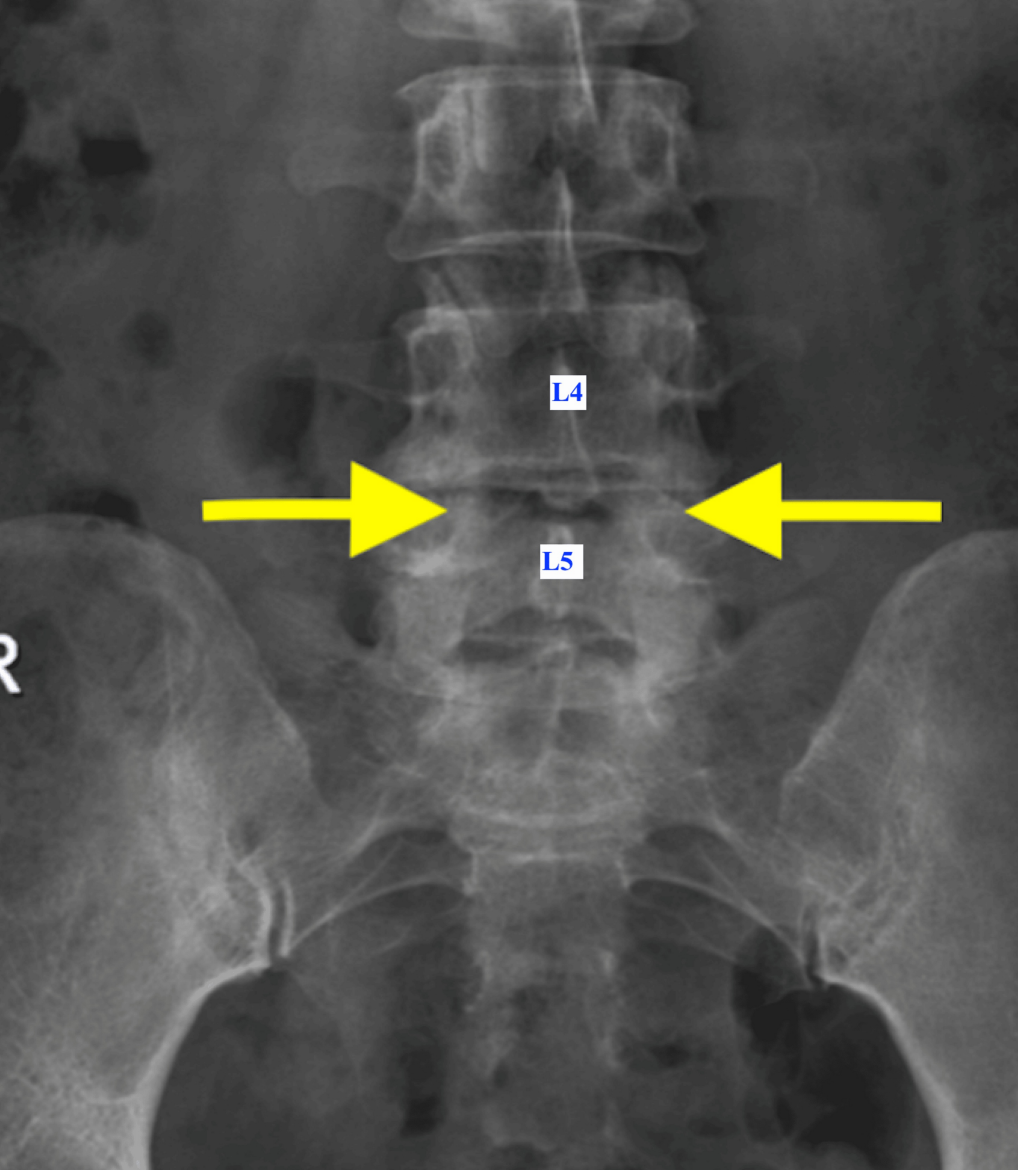
X-ray AP view of the lumbosacral spine showing facet joint arthritic changes at the L4-L5 level (yellow arrows) AP: anteroposterior

**Figure 4:**
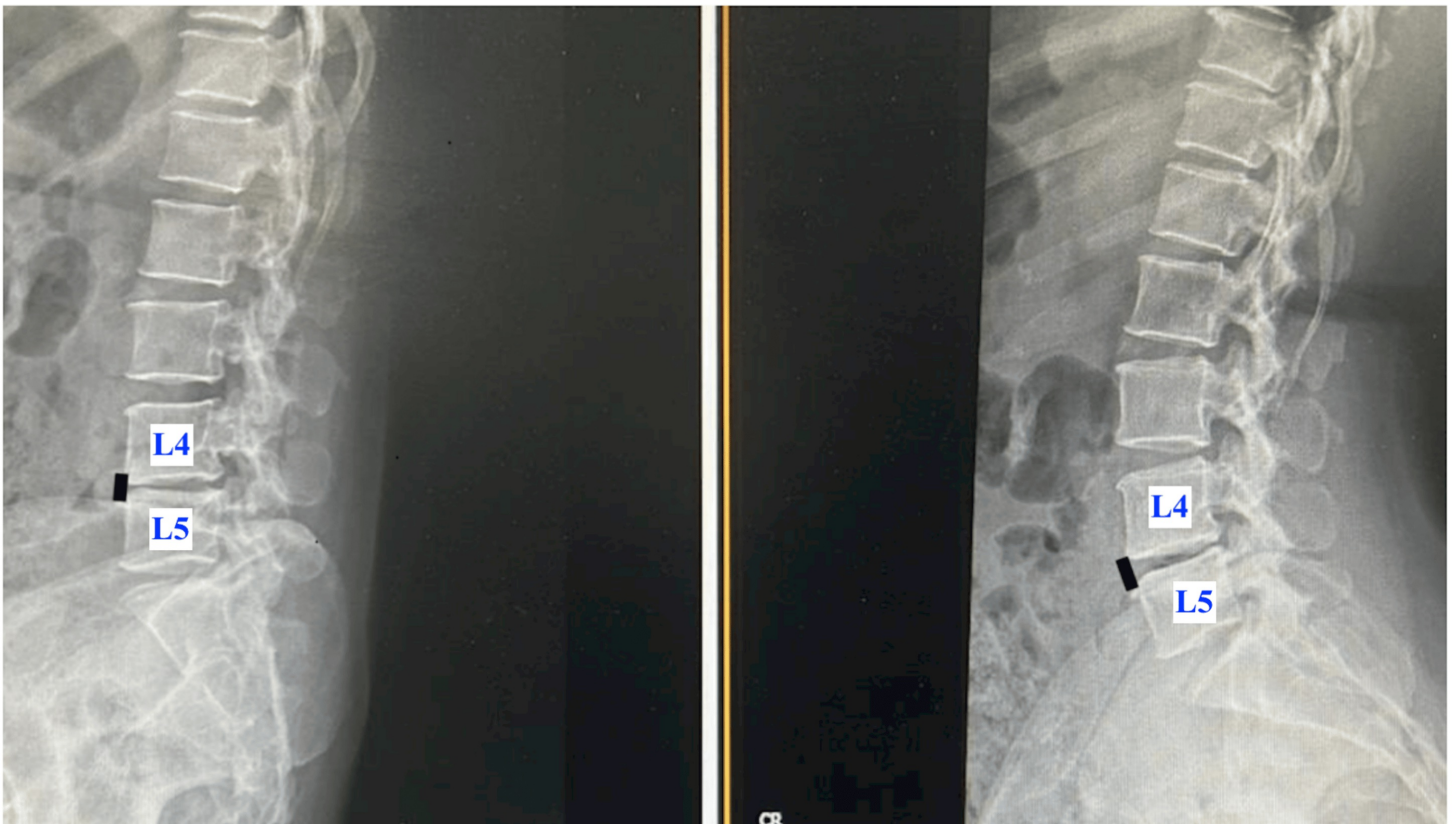
X-ray views of the lumbosacral spine showing instability at the L4-L5 level (A) X-ray lateral view of the lumbosacral spine in flexion showing the closing of the disc space anteriorly (black line) and the opening of the disc space posteriorly at the L4-L5 level. (B) X-ray lateral view of the lumbosacral spine in extension showing the opening of the disc space anteriorly (black line) and the closing of the disc space posteriorly at the L4-L5 level. AP: anteroposterior

**Figure 5:**
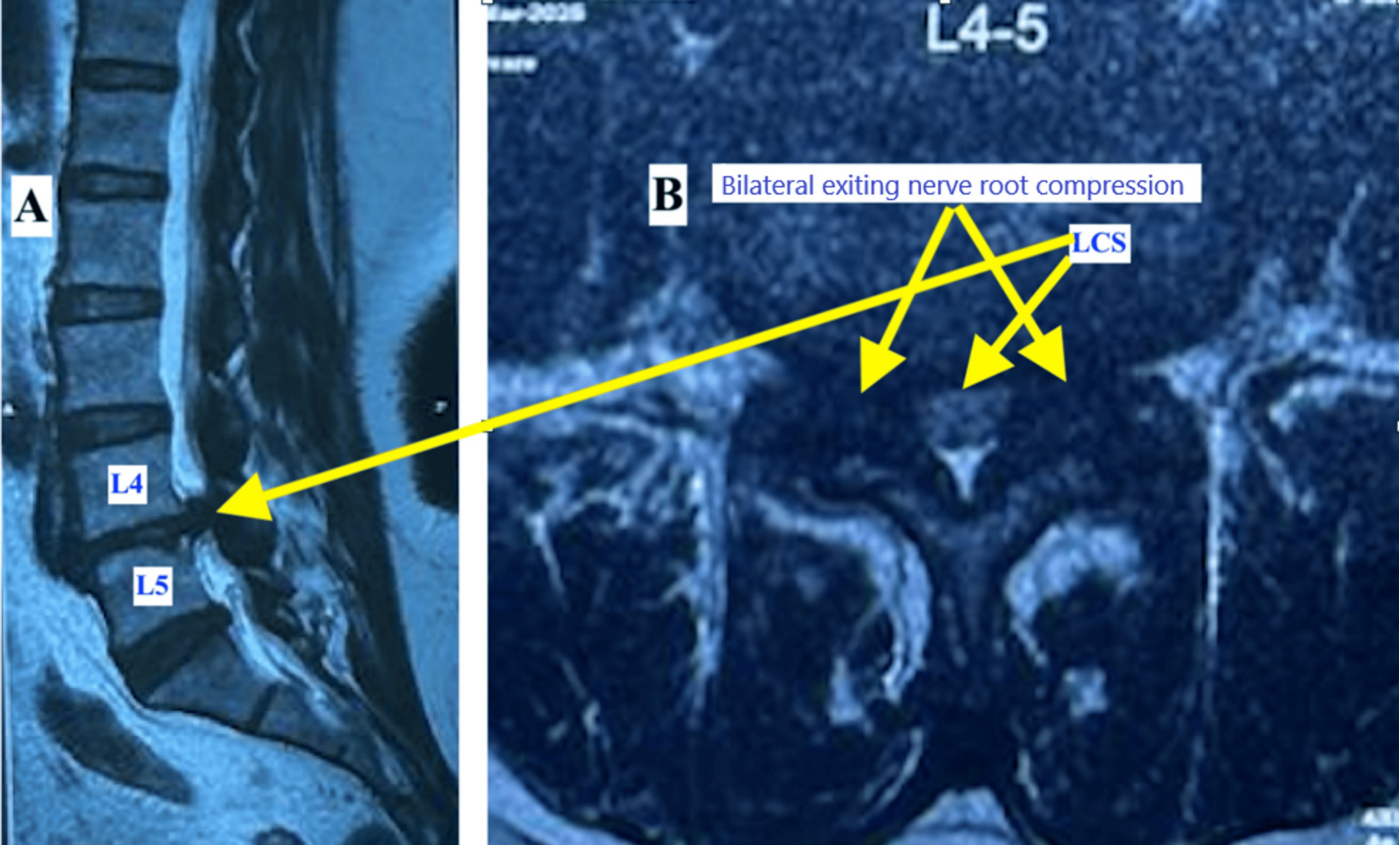
T2-weighted MR images showing lumbar canal stenosis and nerve root compression at the L4-L5 level (A) T2-weighted MR image (sagittal section) showing lumbar canal stenosis at the L4-L5 level (yellow arrow). (B) T2-weighted MR image (axial section) showing bilateral exiting nerve root (L4) compression (yellow arrow). MR: magnetic resonance; LCS: lumbar canal stenosis

Two months after undergoing TKR, the patient presented to our spine surgery service for further evaluation. On clinical examination, she reported neurogenic claudication with a walking distance of approximately 20 meters. The straight leg raise test was positive at 70° bilaterally. There were no clinical signs suggestive of knee infection, although mild tenderness over the lumbar spine was noted. Both knees appeared stiff, likely secondary to inadequate postoperative physiotherapy resulting from pain and muscle weakness during ambulation. Radiographs of both knees demonstrated well-aligned prosthetic components without evidence of loosening or other mechanical complications. Plain radiographs of the lumbosacral spine revealed instability with facet joint arthropathy at the L4-L5 level (Figures 3-4). Magnetic resonance imaging (MRI) showed LCS and bilateral foraminal stenosis at L4-L5, correlating with her clinical findings (Figure 5).

**Figure 6:**
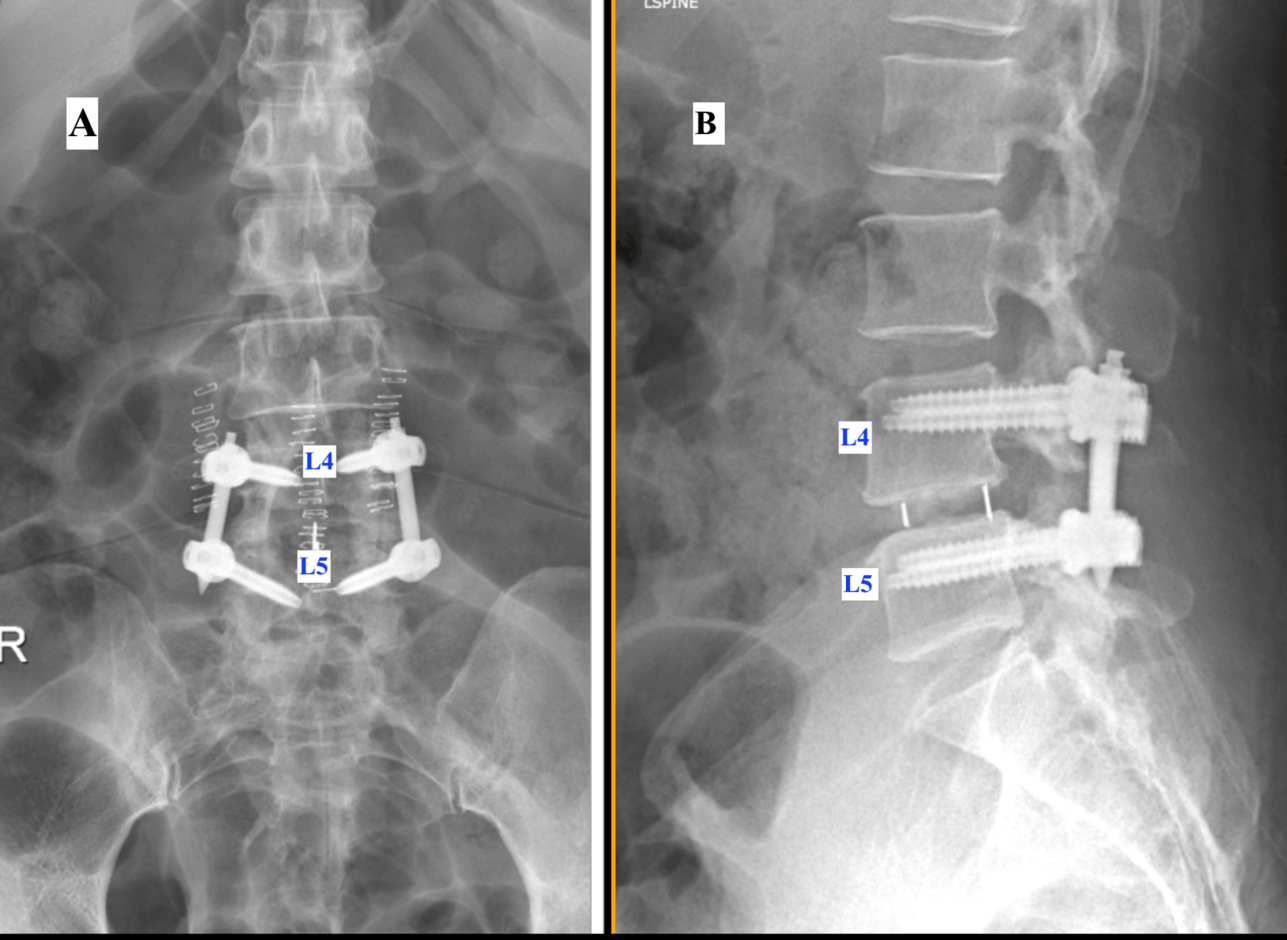
X-ray views of the lumbosacral spine showing pedicle screw fixation and interbody fusion at the L4-L5 level (A) X-ray AP view of the lumbosacral spine showing pedicle screw fixation at the L4-L5 level with interconnecting rod and cage in the disc space. (B) X-ray lateral view of the lumbosacral spine showing pedicle screw fixation at the L4-L5 level with interconnecting rod and cage in the disc space. AP: anteroposterior

The patient underwent lumbar canal decompression and transforaminal lumbar interbody fusion, after which there was significant improvement in her bilateral leg pain and neurogenic symptoms (Figure 6).

Knee stiffness, however, persisted due to delayed rehabilitation, though gradual improvement was observed with dedicated physiotherapy (Table 1).

**Table 1:** Improvement in walking distance and VAS score after addressing the spine pathology Before TKR, the patient had claudication pain after walking for 10 meters. After TKR and one month of proper physiotherapy, this improved to 20 meters, but after addressing the spine pathology, it improved to more than 500 meters. Lower limb pain was quantified using VAS. Before TKR, the patient had excruciating lower limb pain (VAS score: 8). After TKR, the pain reduced to intense type pain (VAS score: 6), but after addressing the spine pathology, there was significant pain reduction (VAS score: 2). TKR: total knee replacement; VAS: Visual Analog Scale

	Before TKR	1 month after TKR	1 month after spine surgery
Walking distance	10 meters	20 meters	More than 500 meters
VAS score	8	6	2

## Discussion

This case highlights the importance of recognizing LCS as a cause of persistent lower limb pain following TKR. Coexisting lumbar degenerative disease is common in elderly individuals and may contribute significantly to postoperative dissatisfaction if not addressed preoperatively.

Knee osteoarthritis and LCS share overlapping symptoms, including lower limb pain, reduced mobility, and functional impairment. Several authors have emphasized the diagnostic challenge in differentiating knee-origin pain from neurogenic claudication [7,8]. In this case, the absence of prominent back pain and failure to focus on claudication further contributed to the underrecognition of spinal pathology.

Persistent postoperative pain after TKR occurs in 10-20% of patients, even though there are various reasons for the same and spinal pathology is a recognized contributing factor [9]. In this scenario, it was confirmed with MRI, which showed LCS with significant nerve root compression at the L4-L5 level. LCS limits walking tolerance, reduces participation in physiotherapy, and can hinder knee rehabilitation. Patients with untreated significant LCS show poorer functional recovery after TKR due to compromised gait mechanics and endurance [10].

Lumbar decompression surgery is effective in alleviating neurogenic claudication and improving function in patients with significant stenosis [11]. In our patient, decompression and fusion of the L4-L5 level resulted in the dramatic relief of leg symptoms, confirming that lumbar pathology, not prosthetic failure, was the primary driver of persistent postoperative pain.

LCS at the L4-L5 level may result in the compression of the traversing or exiting nerve roots, most commonly affecting the L4 nerve root in the lateral recess or foraminal region. This neural compression produces radiculopathy characterized by pain radiating along the corresponding dermatome, often extending to the anterior thigh and knee, thereby mimicking primary knee pathology [1,3]. Such presentation highlights the well-recognized phenomenon of referred knee pain in lumbar spine disorders, emphasizing the importance of careful neurological evaluation in patients presenting with isolated knee symptoms, as spinal pathology may mimic primary knee disease and lead to diagnostic challenges [12,13].

Moreover, the knee and spine function as a biomechanical unit within the lower kinetic chain [14]. Pain, deformity, or stiffness in the lumbar spine can alter gait mechanics, weight-bearing patterns, and overall limb alignment [15]. When such spinal conditions remain undiagnosed or untreated, several postoperative issues may arise following TKR.

Even after the diagnosis of severe knee osteoarthritis has been confirmed and the patient is deemed an appropriate candidate for TKR, a thorough clinical evaluation of the spine remains an essential step in preoperative planning. This is because unrecognized or coexisting spinal pathology, most commonly lumbar degenerative disease, spinal stenosis, or radiculopathy, can significantly influence both the functional outcome and patient satisfaction after TKR.

## Conclusions

In the current era of highly subspecialized orthopedic practice, limited holistic evaluation may lead to missed or misdiagnosed primary conditions. Persistent lumbar spine pathologies, including L3-L4 nerve root compression and altered spinopelvic biomechanics, may negatively influence functional outcomes following TKR. Hence, a focused clinical assessment of the spine, simple, quick, and inexpensive, is essential especially in patients with atypical clinical symptoms. Identification and appropriate management of coexisting spinal pathology prior to TKR may help improve overall functional outcomes. Although larger clinical studies are required to further validate these observations, incorporating routine spine evaluation into the preoperative assessment of patients undergoing TKR, especially in patients with atypical clinical symptoms, may represent an important step toward optimizing surgical outcomes. 
